# Methodological comparison of alpine meadow evapotranspiration on the Tibetan Plateau, China

**DOI:** 10.1371/journal.pone.0189059

**Published:** 2017-12-13

**Authors:** Yaping Chang, Jie Wang, Dahe Qin, Yongjian Ding, Qiudong Zhao, Fengjing Liu, Shiqiang Zhang

**Affiliations:** 1 State Key Laboratory of Cryospheric Sciences, Northwest Institute of Eco-Environment and Resources, Chinese Academy of Sciences, Lanzhou, China; 2 University of Chinese Academy of Sciences, Beijing, China; 3 State Key Laboratory of Hydrology-Water Researches and Hydraulic Engineering, Hohai University, Nanjing, China; 4 Key Laboratory of Ecohydrology of Inland River Basin, Chinese Academy of Sciences, Lanzhou, China; 5 School of Forest Resources and Environmental Science, Michigan Technological University, Houghton, Michigan, United States of America; 6 Shaanxi Key Laboratory of Earth Surface System and Environmental Carrying Capacity, Northwest University, Xi’an, Shaanxi Province, China; 7 College of Urban and Environmental Science, Northwest University, Xi’an, Shaanxi Province, China; Indiana University Bloomington, UNITED STATES

## Abstract

Estimation of evapotranspiration (ET) for alpine meadow areas in the Tibetan Plateau (TP) is essential for water resource management. However, observation data has been limited due to the extreme climates and complex terrain of this region. To address these issues, four representative methods, Penman-Monteith (PM), Priestley-Taylor (PT), Hargreaves-Samani (HS), and Mahringer (MG) methods, were adopted to estimate ET, which were then compared with ET measured using Eddy Covariance (EC) for five alpine meadow sites during the growing seasons from 2010 to 2014. And each site was measured for one growing season during this period. The results demonstrate that the PT method outperformed at all sites with a coefficient of determination (R^2^) ranging from 0.76 to 0.94 and root mean square error (RMSE) ranging from 0.41 to 0.62 mm d^-1^. The PM method showed better performance than HS and MG methods, and the HS method produced relatively acceptable results with higher R^2^ (0.46) and lower RMSE (0.89 mm d^-1^) compared to MG method with R^2^ of 0.16 and RMSE of 1.62 mm d^-1^, while MG underestimated ET at all alpine meadow sites. Therefore, the PT method, being the simpler approach and less data dependent, is recommended to estimate ET for alpine meadow areas in the Tibetan Plateau. The PM method produced reliable results when available data were sufficient, and the HS method proved to be a complementary method when variables were insufficient. On the contrary, the MG method always underestimated ET and is, thus, not suitable for alpine meadows. These results provide a basis for estimating ET on the Tibetan Plateau for annual data collection, analysis, and future studies.

## Introduction

Evapotranspiration (ET) is a significant component in land surface processes related to the water and energy balance and carbon cycles [1]. Traditionally, ET was measured with the micro-lysimeter, Bowen ratio, Scintillometer and pan evaporation methods, while the Eddy Covariance (EC) method has been widely implemented since the turn of the last century [2–5]. Due to the complexity of terrain and climatic conditions over mountains, the number of observation stations is usually very limited, making it difficult to accurately estimate ET in mountainous regions [6], especially in high-altitude mountains.

Over the past decades, many efforts have been made to estimate ET using observations and models in cold climates and high-altitude mountains [7–10], especially in the Tibetan Plateau [11–20]. Currently, there are two primary methods generated for this purpose: remote sensing models, and methods using reference ET. Remote sensing models, such as the Surface Energy Balance Algorithm for Land (SEBAL), Surface Energy Balance System (SEBS), and Mapping Evapotranspiration at high Resolution with Internalized Calibration (METRIC), are commonly used to obtain large-scale terrestrial ET [21–23]. However, it is difficult to acquire land surface variables using these models, which include land surface temperature (LST), normalized difference vegetation index (NDVI), and leaf area index (LAI), with high temporal resolution and also to capture variations of ET over time. And sub-pixel terrain complexity is a major issue with the use of remotely sensed models in the mountains.

Methods that implement a reference ET [24, 25] are commonly used to calculate long-term ET. The standard conditions of reference ET have been defined as a crop height of 12 cm, fixed surface resistance of 70 s m^-1^, and albedo of 0.23 [26], while actual ET is calculated as the product of reference ET and scaling factor (Kc). Allen et al. [26] developed Kc values for different crops under varied climates. Because the Kc value for one crop may change with climate, soil moisture, and growing stage [27], it is necessary to study Kc for different crops in various climates to better quantify ET.

Numerous methods are available for reference ET estimation [25, 28], which can be divided into four categories: combination-based, radiation-based, temperature-based, and mass transfer-based. The water-balance method is another tool to calculate reference ET [29, 30]. Numerous studies have analyzed the performances of these methods, which vary based on different climates [31, 32]. Zhao et al. [33] calculated reference ET using Behnk-Maxey, Priestley-Taylor (PT), and Hargreaves-Samani (HS) methods and found that HS performed best in semi-arid regions in China. Tabari [34] used four equations (Makkink, HS, Turc, and PT equations) to estimate monthly reference ET in four different climate zones in Iran and found that the Turc equation was most suitable for cold humid and arid climates, while the HS equation proved to be the best for warm humid and semi-arid climates. Further, Sabziparvar et al. [35] pointed out that the Orang [36] and Snyder [37] equations outperformed for cold semi-arid and warm arid climates, respectively.

The Tibetan Plateau (TP) is commonly known as the world’s Third Pole due to its extreme climates [38]. Numerous rivers, especially the inland rivers in northwest China, originate from the TP [9, 39], while alpine meadows account for about 15% of China’s cold region that is mainly located in the TP [9]. Therefore, studies on the ET of the TP’s alpine meadows are highly important for understanding the water cycle and energy balance processes. An accurate ET estimation is also a key issue for water resource management and planning [39, 40].

The objective of this study was to evaluate the performance of Penman-Monteith (PM), PT, HS, and Mahringer (MG) methods for ET estimation, which were then compared to EC measurements during the growing season of alpine meadows in the TP, China. These methods were chosen as representatives for each category, previously designated as combination-based, radiation-based, temperature-based, and mass transfer-based. The goal of our study was to find a best method that can be applied as a benchmark for alpine meadows with scarce data over the entire TP area in future studies.

## Materials and methods

### Data at study sites

**Fig 1:**
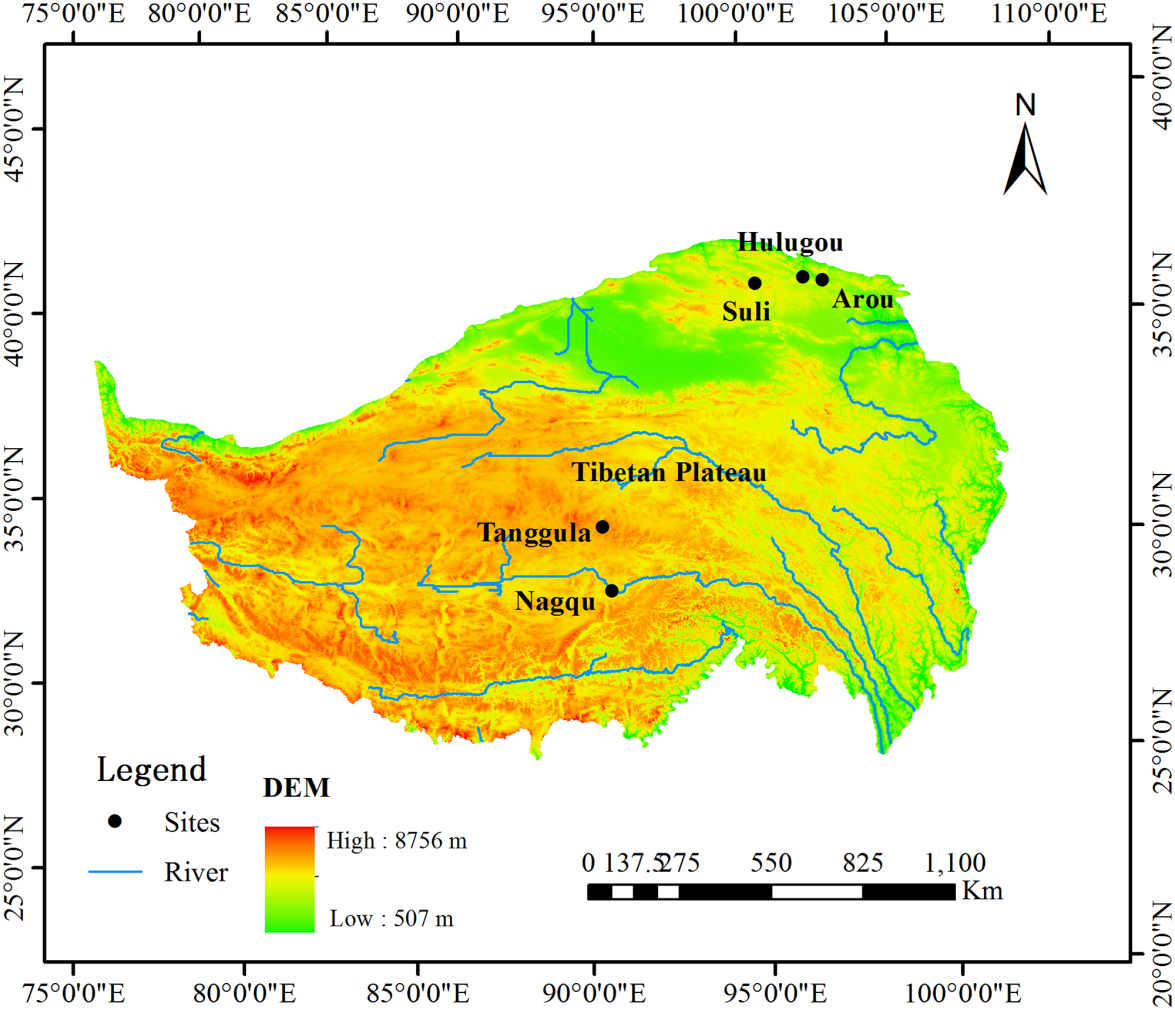
Map of sites with observed flux data in the Tibetan Plateau.

Five flux tower sites, including Arou (38.05°N, 100.46°E; Elevation 3033 m), Hulugou (38.25°N, 99.88°E; Elevation 3232 m), Suli (38.42°N, 98.32°E; Elevation 3885 m), Nagqu (31.37°N, 94.90°E; Elevation 4509 m) and Tanggula (33.07°N, 91.93°E; Elevation 5100 m), were chosen in our study and these sites were established and monitored by the Northwest Institute of Eco-Environment and Resources (NIEER), Chinese Academy of Sciences (CAS) (Fig 1). The NIEER, CAS provided permission for the study to collect field data at each of the sites. Field data (including meteorological and flux data) at five sites (S1 Table), which were available from the NIEER, CAS, were selected to calculate ET using the four estimation methods. The measured ET from the EC tower was used for validation. Measured air temperature, relative humidity, wind speed, net radiation, and soil heat flux were taken every 30 minute and then integrated into daily values. The mean annual temperatures at the Arou, Hulugou, Suli, Nagqu, and Tanggula sites for one single observation year were 0.9, -3.1, -4.0, -0.6, and -4.1°C, respectively. The analyses for the five sites were listed in order from low to high elevation. The mean annual precipitation readings at the Arou, Hulugou, Suli, Nagqu, and Tanggula sites for one single observation year were 403, 553.3, 388.2, 449.5, and 538.2 mm, respectively. At all sites, the land covers were all alpine meadows with semi-arid climates, where elevation ranges from 3033 to 5100 m a.s.l. The information of each site is listed in detail in Table 1. All data were collected during one growing season at each site due to lack of data availability. Due to the only one year of the meteorological data for each site, the mean annual meteorological data (including air temperature and precipitation) of Chinese national stations Tuole and Tuotuohe, which are near the Suli and Tanggula sites, respectively, were used for analysis. The difference of mean annual temperatures of Tuole and Tuotuohe during 2010 with that the five-year (2010–2014) were only -0.1 and 0.6°C, while the annual precipitation difference was 2.6% and 6.7%, which indicates that the influence of the data collected during different years for ET comparison is little.

**Table 1:** Information of sites with observed flux data in the Tibetan Plateau.

Site	Latitude (°)	Longitude (°)	Elevation (m)	Mean annual temperature (°C)	Mean annual precipitation (mm)	Growing season
Arou	38.05	100.46	3033	0.9	403	2014.4.22–2014.10.10
Hulugou	38.25	99.88	3232	-3.1	553.3	2013.4.14–2013.10.14
Suli	38.42	98.32	3885	-4.0	388.2	2010.5.7–2010.9.29
Nagqu	31.37	91.90	4509	-0.6	449.51	2011.4.27–2011.10.21
Tanggula	33.07	91.93	5100	-4.1	538.2	2010.5.17–2010.9.26

The raw EC data were processed by the EdiRe software (University of Edinburgh, http://www.geos.ed.ac.uk/abs/research/micromet/EdiRe). The processing procedures included the removal of spikes, coordinate rotation (3-D rotation), frequency response correction, sonic virtual temperature correction, and corrections for density fluctuation (Webb-Pearman-Leuning, WPL-correction). The raw sampling frequency was 10 Hz. The EC data were averaged with 30-min periods, and data quality assessment was performed for the 30-min EC values using turbulence stationary and integrated turbulence characteristics tests. The 30-min EC data were rejected when precipitation occurred within 1 h before and after data collection and the friction velocity was below 0.1 m s^-1^ at night. The missing data were interpolated by a nonlinear regression method [41, 42].

While EC has been recognized as the most accurate ET estimation method [5, 43–45], an energy closure problem arises when using this method [46, 47], where the turbulent flux (sensible and latent heat flux) is usually less than the available energy [39]. This issue has also been found at observation sites on the TP [39, 48]. The energy balance closure ratios (H+LE) /(R_n_-G) at Arou, Hulugou, Suli, Nagqu, and Tanggula during the growing season were 1.04, 0.67, 0.82, 0.93, and 1.02, respectively. Therefore, a correcting method was applied to address the closure issue, as proposed by Twine et al. [49]:

$$ET=\frac{\left(R_{n}-G\right)}{H_{ori}+LE_{ori}}\times ET_{ori}$$ (1)

where *ET* is the corrected evapotranspiration; *R*_*n*_ is net radiation; *G* is soil heat flux; and *H*_*ori*_, *LE*_*ori*_, and *ET*_*ori*_ are the original sensible heat flux, latent heat flux, and evapotranspiration, respectively. If the number (N) of reliable 30-min measurements was less than 40 per day, the daily measurements were set as null values. Due to the absence of observed meteorological or EC data, the number of available days for Arou, Hulugou, Suli, Nagqu, and Tanggula during each growing season were 80, 117, 146, 149, and 100, respectively.

### Methods for reference ET

#### Combination-based category: FAO PM method

The combination-based method is made up of the available energy and aerodynamic components and has been found to outperform for a variety of crops and climates compared to other methods [28]. Rahimikhoob et al. [50] suggested that the Food and Agriculture Organization (FAO) PM was the best model for this method type. Therefore, PM was selected as the representative method for the combination-based category in this study, which utilizes the following equation:

$$ET_{0}=\frac{0.408\Delta (R_{n}-G)+\gamma \frac{900}{T\;+\;273}u_{2}(e_{s}-e_{a})}{\Delta +\gamma (1+0.34u_{2})}$$ (2)

where *ET*_*0*_ is reference ET; *R*_*n*_ is net radiation (MJ mm d^-1^); *G* is soil heat flux (MJ mm d^-1^); *T* is air temperature (°C); *u*_*2*_ is wind speed at the height of 2 m; *e*_*s*_ is saturated water vapor pressure (kPa); *e*_*a*_ is actual water vapor pressure (kPa); *e*_*s*_*-e*_*a*_ represents the water vapor pressure deficit at reference height (kPa); Δ is the slope of saturated water vapor pressure curve (kPa°C^-1^); and *γ* is the psychrometric constant (kPa°C^-1^).

#### Radiation-based category: PT method

The radiation-based category mainly depends on the available energy and ignores the influence of the aerodynamic component. PT is the most popular method for this category, which integrates an empirical coefficient to avoid the influence of surface and aerodynamic resistances compared to the PM method [51]. The equation for this method is as follows:

$$ET_{0}=\alpha \frac{\Delta }{\Delta +\gamma }(R_{n}-G)$$ (3)

where *α* is Priestley-Taylor coefficient. According to Priestly and Taylor [51] and Rahimikhoob et al. [50], we used *α* = 1.26 in the respective equation in this work.

#### Temperature-based category: HS method

The temperature-based category is empirical and only considers air temperature as a representative of the available energy for ET. It ignores the influence of solar radiation and wind speed, which most likely causes poor results compared to other categories. In this study, we used the HS method [52] for this category, which is described by the following equation:

$$ET_{0}=0.0023{\left(T_{\text{max}}-T_{\text{min}}\right)}^{0.5}(T_{mean}+17.8)R_{a}$$ (4)

where *T*_*max*_, *T*_*min*_, and *T*_*mean*_ are the daily maximum, minimum, and average air temperatures (°C), respectively; and *R*_*a*_ is the water equivalent of extraterrestrial radiation (mm d^-1^).

#### Mass transfer-based category: MG method

The mass transfer-based category is based on Dalton’s gas law, which incorporates wind speed and humidity as parameters. Several equations represent this category, including the Dalton [53], Rohwer [54], Penman [55], and Mahringer [56] equations. We decided to adopt the equation proposed by Mahringer [56] (MG) in this study to analyze the mass transfer-based category:

$$ET_{0}=0.15072\sqrt{3.6u_{2}}(e_{s}-e_{a})$$ (5)

### Determination of scaling factor (Kc)

**Fig 2:**
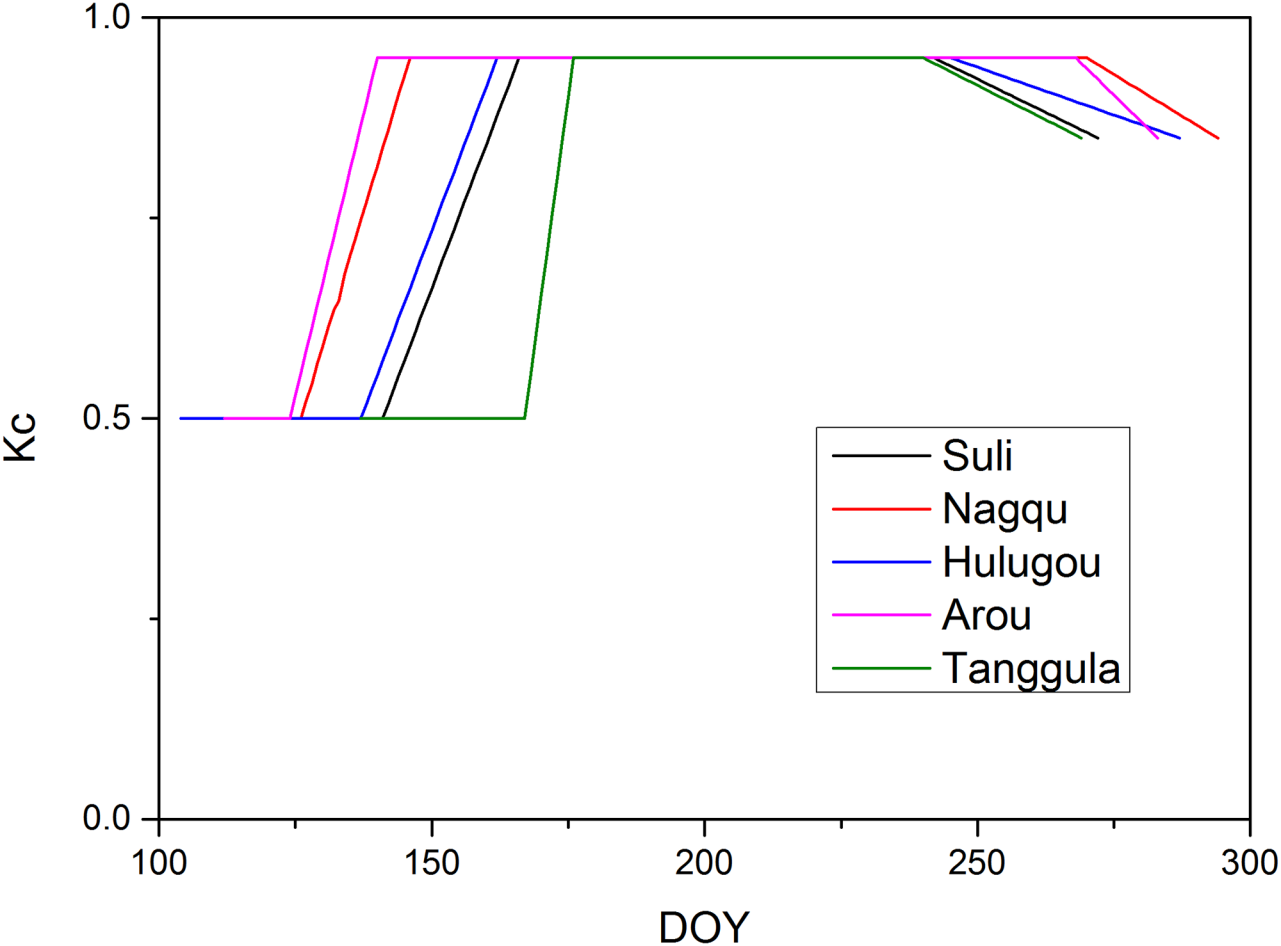
Kc values at different growing periods at 5 sites in the Tibetan Plateau.

Actual ET can be determined by multiplying the reference ET and Kc, which shows that the accuracy of Kc is largely influential. In general, Kc values depend on the growth stages, their length, soil moisture, and leaf area index (LAI). In the FAO Irrigation and Drainage Paper 56, the Kc values in each growth stage for different crops under different climates were defined [26]. The growth stage consists of the initial, development, middle, and end stages. When the mean air temperature of 5 days is greater than 0°C, the alpine meadow enters the initial stage. When the air temperature is consistently greater than 3°C, the alpine meadow begins the development stage. The middle stages begins when the air temperature is greater than 5°C. The end stage occurs when the air temperature is below 5°C, in which the alpine meadow stops growing until the air temperature drops 0°C [57, 58]. In this work, we set a value for each growth stage of the alpine meadows (Table 2) based on the reports by Allen et al.[26], Liu et al.[57], and Li et al.[59]. During the development and end growth stages, Kc values varied linearly and can be defined as [26]:

$$Kc_{i}=Kc_{prev}+\frac{i-\sum L_{prev}}{L_{stage}}(Kc_{next}-Kc_{prev})$$ (6)

where *i* is the day number during the growing season; *Kc*_*i*_, *Kc*_*next*_, and *Kc*_*prev*_ are the scaling factors at day *i*, beginning of the next stage, and end of the previous stage, respectively; *L*_*stage*_ is the length of the current stage; and $\sum L_{prev}$ is the sum length of all previous stages. Detailed information about Kc values for different growing periods can be seen in Table 2 and Fig 2.

**Table 2:** Kc values, start date, and end date of each growth stage of alpine meadow over the Tibetan Plateau, China.

Stage	Initial	Development	Middle	End
Kc	0.5	Equation (6)	0.95	Equation (6) until 0.85
Arou	2014.4.22–2014.5.4	2014.5.5–2014.5.19	2014.5.20–2014.9.25	2014.9.26–2014.10.10
Hulugou	2013.4.14–2013.5.17	2013.5.18–2013.6.10	2013.6.11–2013.9.2	2013.9.3–2013.10.14
Suli	2010.5.7–2010.5.21	2010.5.22–2010.6.14	2010.6.15–2010.8.30	2010.8.31–2010.9.29
Nagqu	2011.4.27–2011.5.6	2011.5.7–2011.5.25	2011.5.26–2011.9.27	2011.9.28–2011.10.21
Tanggula	2010.5.17–2010.6.16	2010.6.17–2010.6.24	2010.6.25–2010.8.28	2010.8.29–2010.9.26

### Evaluation criteria

To evaluate the performance of these methods on the alpine meadow, statistical analysis of the coefficient of determination (R^2^), root mean square error (RMSE), mean absolute error (MAE), relative error (RE), mean bias (MB), and Nash-Sutcliffe efficiency coefficient (NSE) was performed. The equations for these variables are described as follows:

$$R^{2}=\frac{{\left[\sum_{i=1}^{n}\left(Q_{est}-\bar{Q_{est}})(Q_{obs}-\bar{Q_{obs}}\right)\right]}^{2}}{\sum_{i=1}^{n}{\left(Q_{est}-\bar{Q_{est}}\right)}^{2}\;\sum_{i=1}^{n}{\left(Q_{obs}-\bar{Q_{obs}}\right)}^{2}}$$ (7)

$$RMSE=\sqrt{\frac{\sum_{i=1}^{n}{\left(Q_{est}-Q_{obs}\right)}^{2}}{n}}$$ (8)

$$MAE=\frac{\sum_{i=1}^{n}\left|Q_{est}-Q_{obs}\right|}{n}$$ (9)

$$MB=\frac{\sum_{i=1}^{n}\left(Q_{obs}-Q_{est}\right)}{n}$$ (10)

$$RE=100\;\sum_{i=1}^{n}\frac{\left(Q_{obs}-Q_{est}\right)}{Q_{obs}}$$ (11)

$$NSE=1-\frac{\sum_{i=1}^{n}{\left(Q_{obs}-Q_{sim}\right)}^{2}}{\sum_{i=1}^{n}{\left(Q_{obs}-\bar{Q_{obs}}\right)}^{2}}$$ (12)

where *Q*_*obs*_ and *Q*_*est*_ are the observed and estimated values, respectively; $\bar{Q_{obs}}$ and $\bar{Q_{est}}$ are the mean values corresponding to *Q*_*obs*_ and *Q*_*est*_, respectively; and *n* is the sample number.

## Results

**Fig 3:**
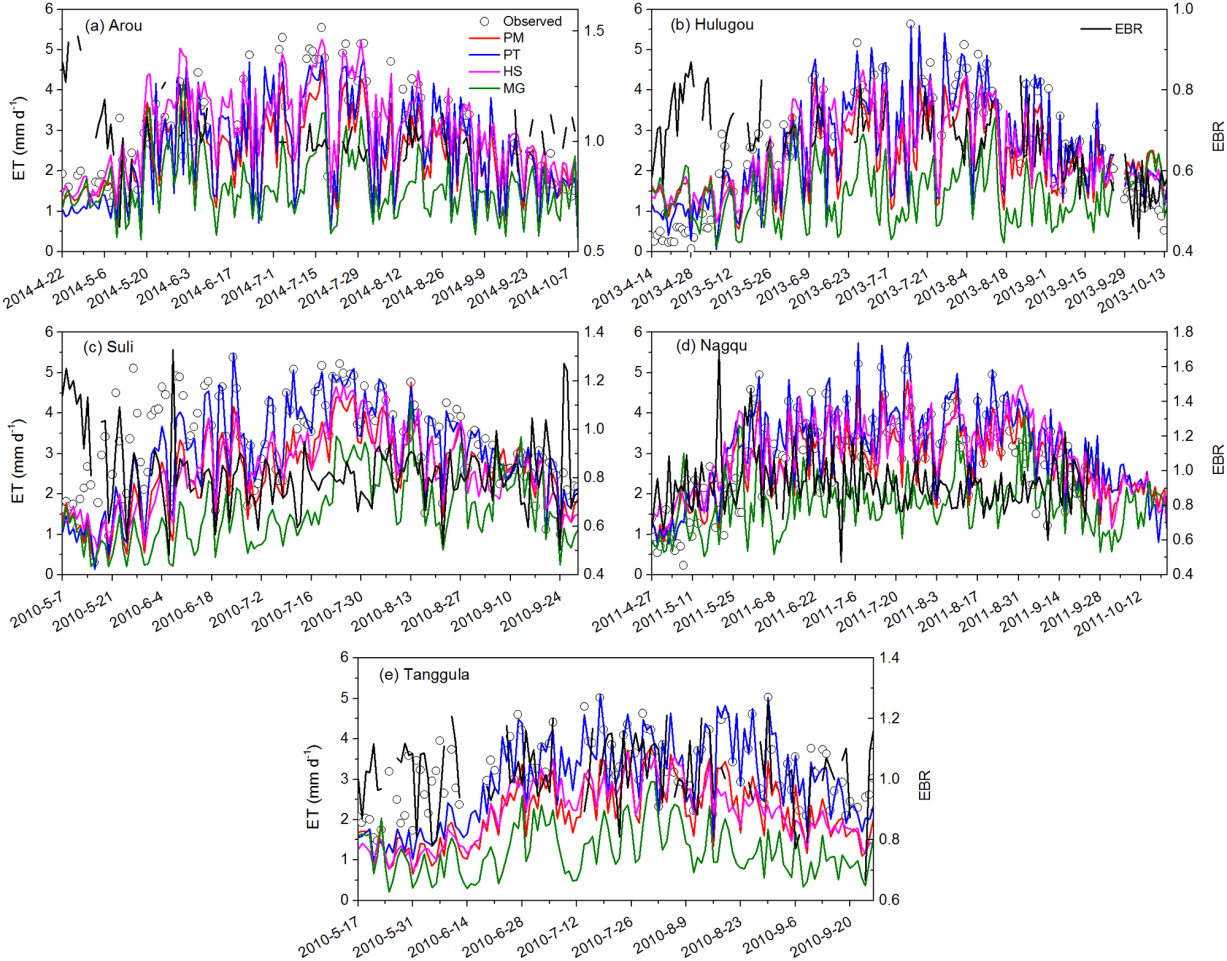
Comparisons of estimated daily ET time series using four methods with ET measured by EC and variation of energy balance closure ratio (EBR)at Arou (a), Hulugou (b), Suli (c), Nagqu (d), and Tanggula (e) sites during the growing season.

**Fig 4:**
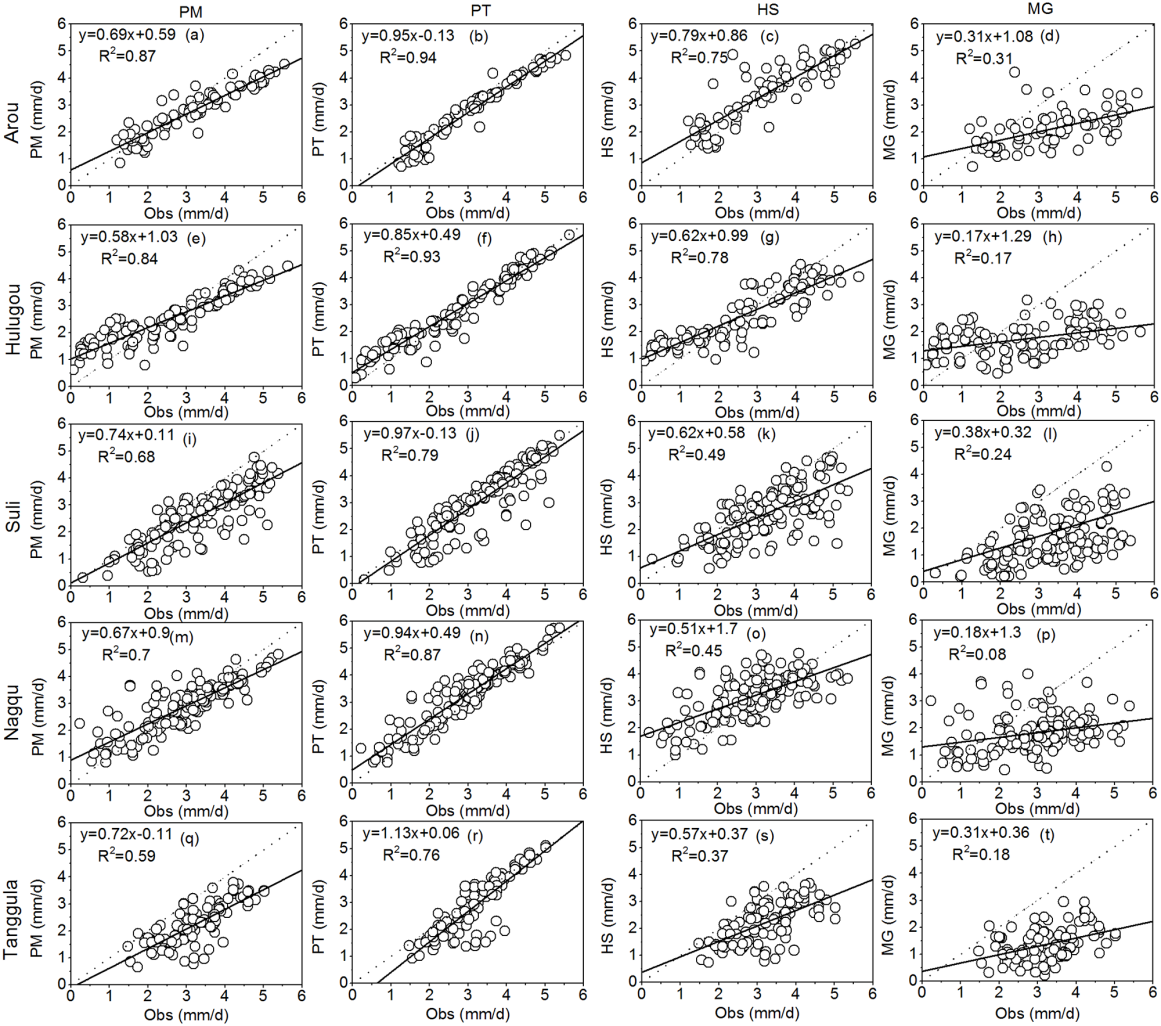
Scatter plots of ET estimated by four methods and measured ET at Arou (a, b, c, d), Hulugou (e, f, g, h), Suli (i, j, k, l), Nagqu (m, n, o, p), and Tanggula (q, r, s, t) sites during the growing season. The black line represents linear regression line, and the dashed line is 1:1 line.

**Fig 5:**
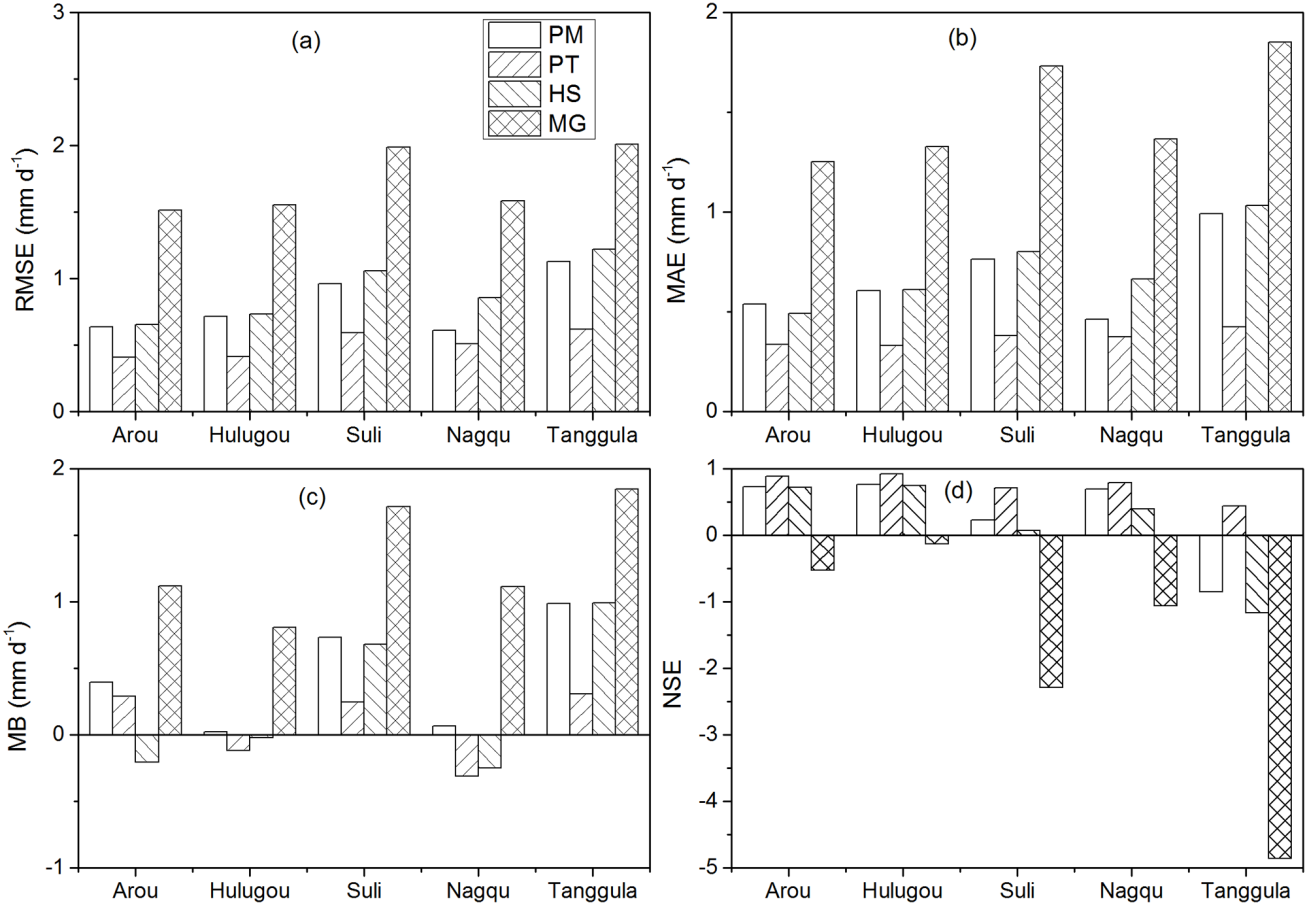
Comparison of statistical indicators (RMSE, MAE, MB and NSE) using the four daily ET estimation methods at Suli, Nagqu, Hulugou, Arou, and Tanggula sites during the growing season.

R^2^ of all sites between ET before and after-correction for Arou, Hulugou, Suli, Nagqu, and Tanggula were 0.92, 0.96, 0.83, 0.82 and 0.86, respectively. And the MAEs between ET before and after-correction for Arou, Hulugou, Suli, Nagqu, and Tanggula were 0.25, 0.81, 0.67, 0.40, and 0.29 mm d^-1^, respectively. We used the measured data after correction as comparison. The time series of daily ET during the growing season estimated from PM, PT, HS, MG, and measured data are shown in Fig 3, and the statistics of ET estimation using the four methods are listed in Table 3. Daily ET estimated by PM, PT, HS, and MG methods over five representative sites were compared to that estimated by EC (Fig 4). Fig 5 presents the statistical analysis of ET estimated by different methods. Based on the results, the PT method revealed the best performance at all stations with the highest R^2^, lowest RMSE, and highest NSE values compared to the other three methods.

**Table 3:** Total ET of observed and estimation of four methods during the whole observation period.

Site	Method	Total measured ET (mm)	Total estimated ET (mm)	RE (%)
Arou	PM	255	223.6	8.9
	PT		232	10.2
	HS		271.6	-11.1
	MG		165.6	28.6
Hulugou	PM	294.7	292.5	-48.2
	PT		308.4	-30
	HS		297.2	-52.4
	MG		200.5	-26.7
Suli	PM	478.1	371.6	21.9
	PT		442.1	8.6
	HS		379.1	17.1
	MG		227.7	51
Nagqu	PM	436.2	426.9	-10.6
	PT		483	-17.4
	HS		473.8	-25.6
	MG		270.7	20.4
Tanggula	PM	320	221.5	30.8
	PT		289.1	11.2
	HS		220.7	30.3
	MG		135.4	56.5

At the Arou site (relative lower elevation), the mean ET of PM, PT, HS, MG, and measured data were 2.79, 2.9, 3.39, 2.07, and 3.19 mm d^-1^, respectively, during the growing season (Fig 3a). ET was underestimated by PM, PT, and MG with RE values of 8.9%, 10.2%, and 28.6%, respectively, while HS overestimated ET with an RE of -11.1%. However, ET estimated by PM, PT, and HS had higher consistencies with measured values, and ET was underestimated via the MG method with a bias of 1.12 mm d^-1^. The PT method performed best with the lowest RMSE (0.41 mm d^-1^), highest NSE (0.89), and lowest bias (0.29 mm d^-1^) values during the growing season in 2014 at Arou station. PM and HS showed reduced performance with similar RMSE (0.64 and 0.65 mm d^-1^) and NSE (0.73 and 0.71) values, while MG presented the worst results with the highest RMSE (1.51 mm d^-1^) and a negative NSE (-0.53).

At the Hulugou site (relative lower elevation), the mean ET of PM, PT, HS, MG, and measured data were determined to be 2.5, 2.64, 2.54, 1.7, and 2.52 mm d^-1^, respectively, during the growing season (Fig 3b). REs of PM, PT, HS and MG were -48.2%, -30%, -52.4%, and -26.7%, respectively (Table 3). ET estimated by the PM, PT, and HS methods had better consistency with measured ET. At the beginning and end of the growing season, all methods overestimated ET with large errors. PT displayed good performance with the highest R^2^ (0.93), lowest RMSE (0.41 mm d^-1^), and highest NSE (0.92). PM and HS showed slightly similar results with similar R^2^ (0.84 and 0.78) and RMSE (0.76 and 0.75 mm d^-1^) values. MG performed the worst with the highest bias (0.81 mm d^-1^), highest RMSE (1.55 mm d^-1^), and a negative NSE (-0.13).

At the Suli site (relative middle elevation), the mean ET of PM, PT, HS, MG, and measured data were 2.55, 3.03, 2.6, 1.56 and 3.27 mm d^-1^, respectively (Table 3). Overall, ET was underestimated by these methods with positive RE values. The PT method exhibited good performance with the highest R^2^ (0.79), lowest RMSE (0.59 mm d^-1^), and highest NSE (0.71) values (Fig 5), suggesting that this method estimated ET best. The second best method was the PM method with R^2^ = 0.68, RMSE = 0.96 mm d^-1^, and NSE = 0.23, which was followed by the HS method with similar RMSE and MAE values but a lower RE. However, NSE of the HS method was close to zero, which indicates poor results, while the MG method performed the worst with the weakest correlation (R^2^ = 0.24), largest bias (1.72 mm d^-1^), and largest RMSE (1.99 mm d^-1^).

At the Nagqu site (relative middle elevation), the whole measured ET during the growing season in 2011 was 436.2 mm, where REs of PM, PT, and HS were -10.6%, -17.4%, and -25.6%, respectively (Table 3). ET was underestimated by the MG method with an RE of 20.4%, which suggests large uncertainties in the method. The PM and PT methods had lower RMSE values (0.61 and 0.51 mm d^-1^, respectively) compared to the HS and MG methods. ET estimated by PT had good consistency with the measured ET (R^2^ = 0.87), while PM had the smallest bias (0.06 mm d^-1^). PT performed the best with the highest R^2^ (0.87), lowest RMSE (0.51 mm d^-1^), and highest NSE (0.79), and PM proved to be the second-best method with a lower RMSE (0.61 mm d^-1^) and higher NSE (0.69). HS showed reduced performance with an RMSE of 0.86 mm d^-1^ and NSE of 0.39, which was followed by PT and PM. MG performed the worst with the highest RMSE (1.58 mm d^-1^) and negative NSE (-1.1) values, which means that MG may be not a suitable method for alpine meadows.

At the Tanggula site (relative higher elevation), the mean ET of PM, PT, HS, MG, and measured data were 2.21, 2.89, 2.21, 1.35, and 3.2 mm d^-1^ during the growing season in 2010 (Fig 3e), where ET estimated by PM and PT had higher correlation with measured ET. However, the REs of PM, PT, HS, and MG were determined to be 30.8%, 11.2%, 30.3%, and 56.5%, respectively (Table 3), which indicates that all methods underestimated ET at the Tanggula site. Among them, the PT method presented the best results with the highest R^2^ (0.76), lowest RMSE (0.62 mm d^-1^), and highest NSE (0.44), while PM showed reduced performance with a higher R^2^ (0.59) and lower RMSE (1.13 mm d^-1^). The HS and MG methods displayed the poorest performance with lower R^2^ (0.37 and 0.18, respectively) and higher RMSE (1.22 and 2.0 mm d^-1^) values. All methods produced a negative NSE, except PT with an NSE of 0.44, suggesting that the PT method performed best at the Tanggula site.

**Fig 6:**
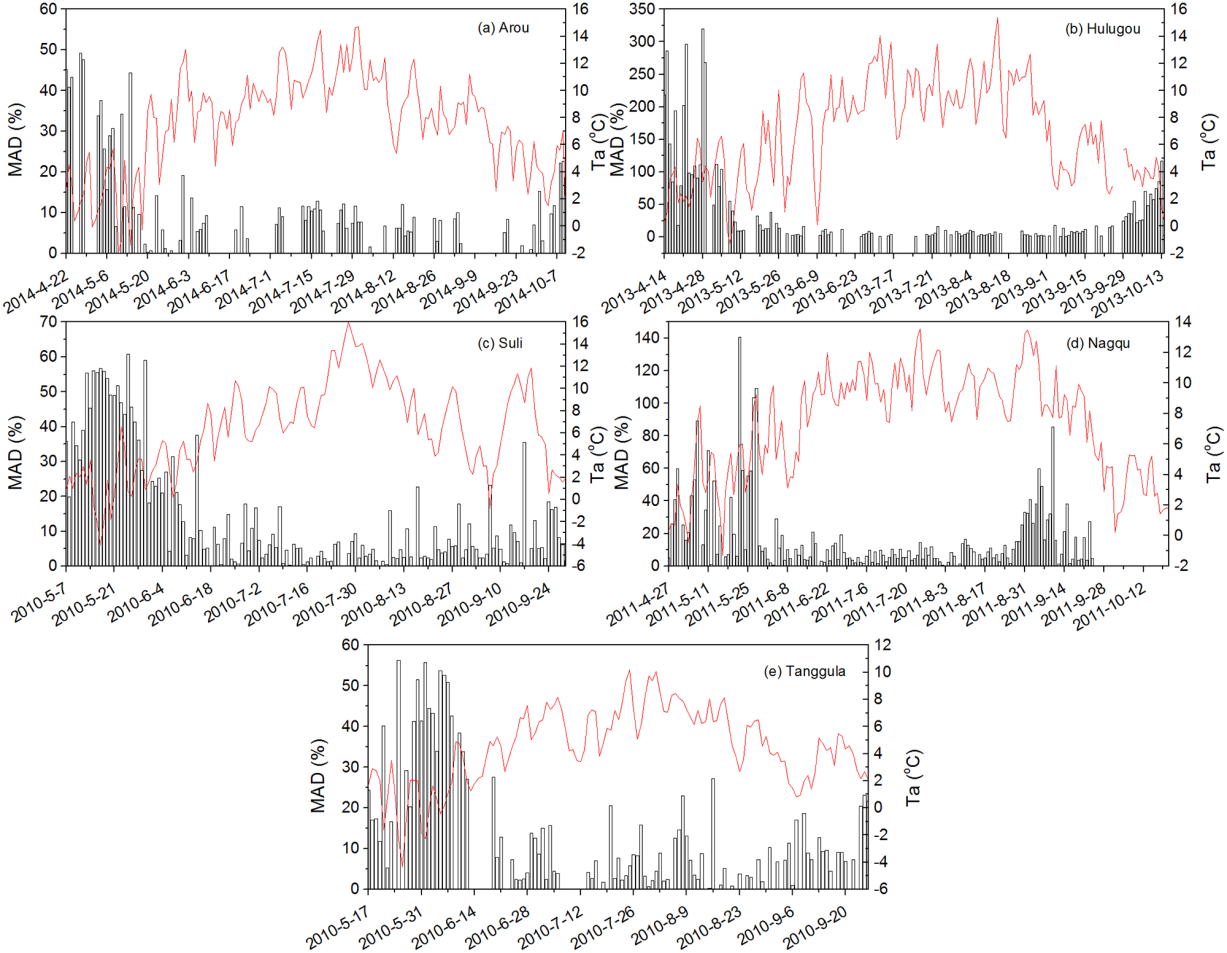
Variation of mean absolute deviation (MAD) and air temperature (Ta) at Arou (a), Hulugou (b), Suli (c), Nagqu (d) and Tanggula (e) sites during the growing season.

Noting that all methods underestimated ET during May to mid-June at the Suli and Tanggula sites (Fig 3c and 3e). Moreover, all methods had large biases at the beginning of the growing season. Thus, we took PT method (the best method as has been shown above) as an example to explore the methods application and limitation on alpine meadow. The mean absolute deviation $\sum_{\text{i}=1}^{\text{n}}\left|\frac{{\text{Q}}_{\text{obs}}{\text{-Q}}_{\text{est}}}{{\text{Q}}_{\text{obs}}}\right|\text{/n}$ (MAD) was chosen as a statistic. PT had large MAD when temperature were lower than 2°C, especially at the initial stage of the growing season (Fig 6). The MAD during the initial stage of the growing season for Arou, Hulugou, Suli, Nagqu and Tanggula sites were 42.5%, 119.3%, 45.2%, 29.1%, and 35.4%, respectively. The MAD of Suli and Tanggula sites during May to mid-June were about 34.4% and 35.4%, which means the meadows growed slowly and the temperature during this period were 2.22 and 1.09°C, respectively. This may be caused by the low temperature at the beginning of the growing season while some days appeared negative temperature which means that snow/ice events might occur, almost all the methods used in this paper could not predict ET of snow/ice surface accurately due to their own limitations. Therefore, all methods cannot capture high accuracy ET of alpine meadow when air temperature was less than 2°C during the growing season, especially at the beginning of the growing season.

In summary, the PT method produced the best results for all stations. The PM and HS methods showed reduced performance for all stations, while the MG method may be not suitable for alpine meadows in the TP.

## Uncertainties and limitations of performance of different methods

Compared with the measured ET before correction, PT performed better with higher R^2^ (0.6) and lower RMSE (0.85 mm d^-1^) than other methods among all sites, while it showed better performance with R^2^ of 0.82 and RMSE of 0.52 mm d^-1^ compared with EC data after correction. Though EC values have been regarded as ‘truth’ values for validation, the uncertainties of the EC data are difficult to present which primarily due to the stochastic error and systematic error [60, 61]. The stochastic error depends on the number of independent observations when time series are auto- or cross-correlated [60]. The systematic error is not only related on the instrument, but also affected by the unmet environmental conditions and the complex procedure for data processing. For the five sites, the daily EBR was 0.93 during single growing season for different years. The systematic error $\delta_{\text{F}}^{\text{sys}}=\text{F}(\frac{\text{1}}{\text{EBR}}-1)$ can be directly calculated from EBR of 30-min during daytime conditions (global radiation > 20 W m^-2^), where *F* means the scalar fluxes. Thus the systematic error of the turbulent fluxes for the five sites was about 10%. Generally, higher EBR would decreased the uncertainty of the measured data. The EBR higher than 1 indicates that the turbulent flux were larger than the available energy which was unreasonable. Similarly, lower EBR would produce large uncertainty due to the energy imbalance. In order to acquire the data close to the “truth” value, the correction for measured data was necessary for energy closure [45]. The correction would decrease measured ET when EBR was higher than 1 and vice versa. However, the correction for energy balance still produced the uncertainty of the performance of different methods.

**Fig 7:**
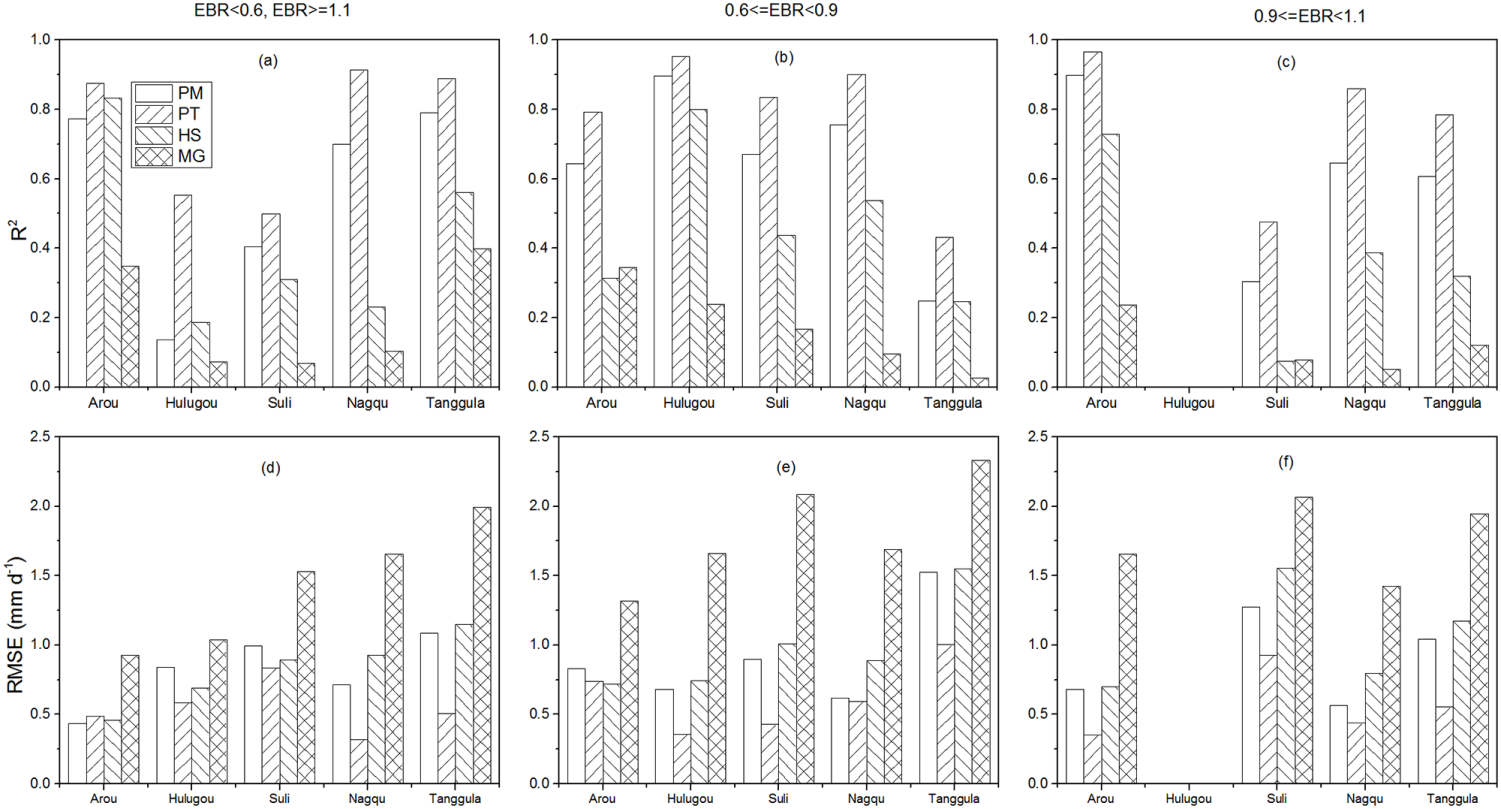
R^2^ and RMSE of the four daily ET estimation methods over different energy balance closure ratio (EBR) intervals at Arou, Hulugou, Suli, Nagqu, and Tanggula sites during the growing season.

Higher MAE between ET before and after-correction corresponding to lower EBR implies the large uncertainty for measured ET. Therefore, the analyses of the performance of different methods over different EBR thresholds are needed (Fig 7). Among all sites, the PT method performed best over all different intervals than other three methods. It performed best with R^2^ of 0.97 and RMSE of 0.35 mm d^-1^ at the EBR interval [0.9, 1.1) than other intervals at the Arou site while the PM method performed similar with the PT method during EBR less than 0.6 or more than 1.1. Though the EBR at the Hulugou site was 0.68, which means large uncertainty of EC data exists, the PT method performed better than other methods over different intervals. And the performance was best during the interval EBR interval [0.6, 0.9). The PT method also performed better with higher R^2^ and lower RMSE over different intervals, followed by the PM method at the Suli, Nagqu, and Tanggula sites. And the MG method performed worst over different intervals. The PT and PM methods performed best during the EBR interval [0.6, 0.9) at the Suli and Tanggula sites. The PT method performed best during the EBR less than 0.6 and more than 1.1 than other intervals at the Nagqu site. The better performance of PT method at the EBR interval [0.6, 1.1) indicates that higher accuracy measured values are necessary for validation. Though the PT method could produce better results than other methods during the EBR less than 0.6 and more than 1.1, both the corrected EC data and the results of the methods have large uncertainties which make meaningless for comparison and validation.

Besides the uncertainty of EC data, several researchers have studied the performance of different methods. Yang et al. [9] considered that the PM method offered better results than the PT method when compared with ET observations using the micro-lysimeter in the Qilian Mountains. Wu et al. [62] found that the PT method showed considerable difference with the PM method, which was used as a reference in the middle of Heihe River. Therefore, the performance of the PM and PT methods may be different on the alpine meadows, while the PM method has been considered the standard method for ET estimation with more rigorous physical meaning [26]. Ma et al. [17, 18] compared the complimentary relationship and PM methods in an alpine steppe of the TP and ascribed that the complimentary relationship and PM methods could provide efficient results using meteorological data. Zhu et al. [63] recommended using the PT method when available data were limited for alpine grassland on the TP.

Moreover, the PM method needs more input variables, which limits its application in data-scarce region [34]. There have limited observation sites on the TP due to the harsh environment [17]. Net radiation and air temperature can be required with spatial interpolation or remote sensing data for the whole TP region while RH and wind speed may have large uncertainties. Unfortunately, it is difficult to obtain high accuracy ET using PM due to limited input data and to analyze the exact specific meteorological condition when the PT outperforms PM under the current available dataset. Future studies will aim at decreasing these difficulties. We also noted that the collected data were come from different years, which will bring some uncertainties on the evaluation of different methods.

**Fig 8:**
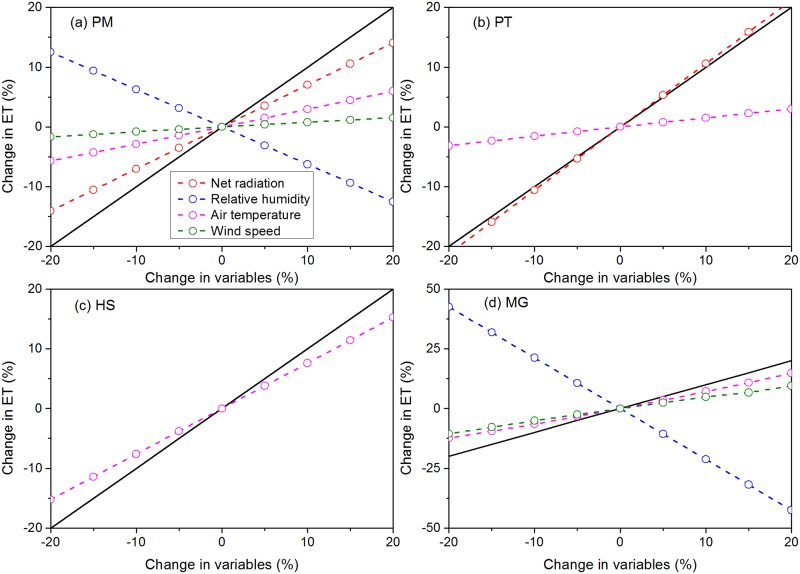
Sensitivity analysis for ET estimated by PM (a), PT (b), HS (c), and MG (d) methods with corresponding input variables.

To obtain the main limiting factor of each method, we calculated the sensitivity coefficient of input variables for each method (Fig 8).

**Fig 9:**
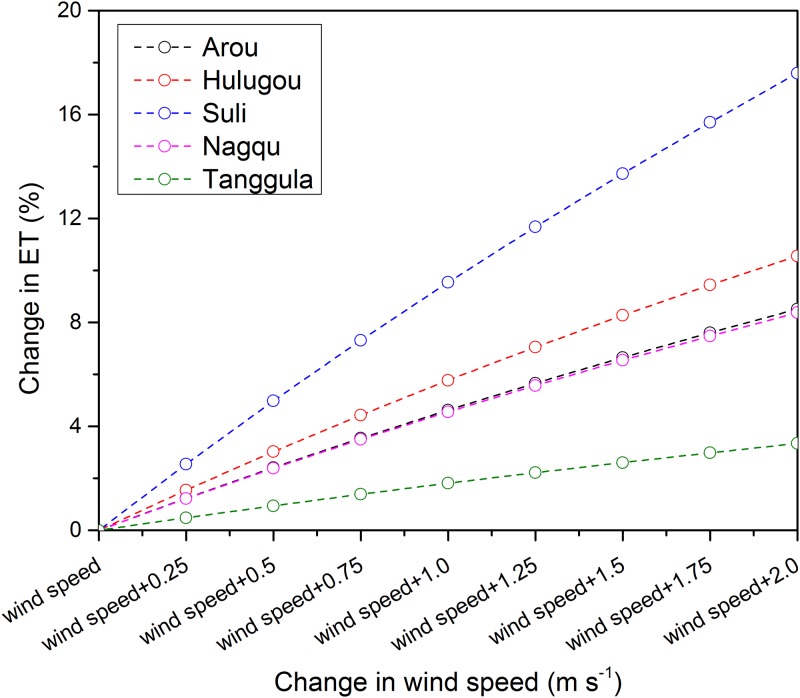
Sensitivity analysis of wind speed by the PM method for each site with different elevations.

The PM method exhibited good performance at Suli and Hulugou sites. In addition to net radiation, relative humidity is another important factor that influences water vapor deficit and the accuracy of ET estimation by the PM method [64]. Air temperature and wind speed were found to have little impact on the PM method (Fig 8a). In order to estimate the influence of wind speed over different elevations, we calculated the sensitivity of wind speed for each site which were located at different elevations (Fig 9). The sensitivity of the wind speed to ET was highest at the Suli site (relative middle elevation), while the sensitivity coefficient was lower at the Tanggula site (relative higher elevation), indicating that wind speed was not sensitive to ET in the high altitude areas which had higher wind speed. Generally, wind speed increased with increasing altitude gradients. Thus, the sensitivity of the wind speed to ET was higher at the lower elevation sites than the higher elevation sites.

It can be concluded that PT performed well at all stations, which may be due to the simple parameterization of PT. The available energy and air temperature were the most influential factors for PT, while ET estimated by PT was not sensitive to air temperature (Fig 8b). The net radiation was a dominant factor of the PT method, and had higher correlation with ET; thus, it was considered in both PM and PT methods. This may be the reason behind the better performance of the PM and PT methods compared to HS and MG [9].

HS showed reduced performance at most alpine meadow sites over the TP, which may be explainable by this method’s simple empirical formula that is mainly based on air temperature and extraterrestrial radiation. Consideration of the available energy and soil moisture would most likely improve the performance of this method. *R*_*a*_ was used rather than incoming solar radiation as radiation data, which does not consider atmospheric transmissivity [34, 65], while air temperature was the only variable that had a large impact on the HS method.

MG was found to underperform compared to all methods, suggesting that it is not suitable for alpine meadow areas. This method does not describe the available energy conditions although air temperature, relative humidity, and wind speed are considered as parameters. Among the three variables, the sensitivity of relative humidity was greater than air temperature and wind speed.

Overall, net radiation had a large impact on the PM and PT methods. RH showed higher sensitivity for PM and MG, and air temperature had the greatest impact on the HS method as the only input variable. Moreover, wind speed was found to have minimal influence on the PM and MG methods.

## Conclusions

We evaluated PM, PT, HS, and MG as ET estimations methods by comparing observed ET via EC at alpine meadow sites on the TP in China. The PM and HS methods showed reduced results, while results for MG suggest that it may not be suitable for most alpine meadow sites. Based on its acceptable results, the PT method is recommended as the most suitable for alpine meadows on the TP to estimate and stimulate the water and energy balance, where sufficient data is not available. The present evaluations may be helpful for deriving the ET over the whole TP, enhancing our understanding of hydrological cycle of Third Pole region. Future work will focus on the estimation of ET for other vegetation cover types to obtain enhanced accuracy ET over the whole TP region.

## Supporting information

S1 Table The meteorological data and flux data of the five sites. The meteorological data and flux data for each site was in the S1 Table.xlsx.
(XLSX)
